# Gene Identification of Pheromone Gland Genes Involved in Type II Sex Pheromone Biosynthesis and Transportation in Female Tea Pest *Ectropis grisescens*

**DOI:** 10.1534/g3.117.300543

**Published:** 2018-01-09

**Authors:** Zhao-Qun Li, Long Ma, Qian Yin, Xiao-Ming Cai, Zong-Xiu Luo, Lei Bian, Zhao-Jun Xin, Peng He, Zong-Mao Chen

**Affiliations:** * Key Laboratory of Tea Biology and Resource Utilization, Ministry of Agriculture, Tea Research Institute, Chinese Academy of Agricultural Science, Hangzhou 310008, People’s Republic of China; † Jiangxi Key Laboratory of Bioprocess Engineering and Co-Innovation Center for In-vitro Diagnostic Reagents and Devices, College of Life Sciences, Jiangxi Science & Technology Normal University, Nanchang 330013, People’s Republic of China; ‡ Institute of Botany, Jiangsu Province and Chinese Academy of Sciences, Nanjing, Jiangsu Province 210014, People’s Republic of China; § State Key Laboratory of Green Pesticide and Agricultural Bioengineering, Ministry of Education, Guizhou University, Guiyang, 550025, People’s Republic of China

**Keywords:** transcriptome, sex pheromone biosynthesis, tissue expression pattern, *Ectropis grisescens*

## Abstract

Moths can biosynthesize sex pheromones in the female sex pheromone glands (PGs) and can distinguish species-specific sex pheromones using their antennae. However, the biosynthesis and transportation mechanism for Type II sex pheromone components has rarely been documented in moths. In this study, we constructed a massive PG transcriptome database (14.72 Gb) from a moth species, *Ectropis grisescens*, which uses type II sex pheromones and is a major tea pest in China. We further identified putative sex pheromone biosynthesis and transportation-related unigenes: 111 cytochrome P450 monooxygenases (CYPs), 25 odorant-binding proteins (OBPs), and 20 chemosensory proteins (CSPs). Tissue expression and phylogenetic tree analyses showed that one CYP (*EgriCYP341-fragment3*), one OBP (*EgriOBP4*), and one CSP (*EgriCSP10*) gene displayed an enriched expression in the PGs, and that *EgriOBP2*, *3*, and *25* are clustered in the moth pheromone-binding protein clade. We considered these our candidate genes. Our results yielded large-scale PG sequence information for further functional studies.

Reproductive isolation in moths relies on species-specific and multiple sex pheromone components, which are diverse in structure and ratio (Ando *et al.* 2004). Sex pheromone components are divided into three types according to their structure: type I (75%), type II (15%), and miscellaneous type (10%) (Ando *et al.* 2004; Löfstedt *et al.* 2016). Type I sex pheromone components comprise a C_10_–C_18_ straight chain with unsaturated aliphatic compounds, and different terminal functional groups making them an alcohol, aldehyde, or acetate. Type II consists of one to three *cis* double bonds separated by methylene groups of C_17_–C_23_ straight chains, in addition to zero, one, or two epoxide functions (Millar 2000; Ando *et al.* 2004).

Type I sex pheromone components are biosynthesized from saturated fatty acids, typically palmitic acid, through many enzymatic reactions (Jurenka 2004a; He *et al.* 2017), whereas type II sex pheromone components are derived through decarboxylation and epoxidation from dietary linoleic or linolenic acid (Jurenka 2004a; Tillman *et al.* 1999; Millar 2000). Production sites also differ between type I and type II. Type I pheromone components are usually produced and released from female extrudable glands (pheromone glands, PGs) located between the eighth and ninth abdominal segments. However, the hydrocarbon precursor of type II pheromone components is expected to be produced from dietary linolenic acid by chain elongation and decarboxylation in oenocytes and then transported to the PG in which it is epoxidized and released (Millar 2000; Jurenka 2003, 2004b). The proteins that are responsible for transportation and epoxidation of the type II sex pheromone precursor are not yet well documented.

Odorant-binding proteins (OBPs) and chemosensory proteins (CSPs) are two types of soluble protein that are well known as odor transporters (including transporters of sex pheromones) in insect antennal lymph (Vogt *et al.* 2015; Pelosi *et al.* 2014). Numerous insect OBPs and CSPs have been identified recently by the blooming sequencing techniques (X.-M. Li *et al.* 2015; He and He 2014; He *et al.* 2011; Xu *et al.* 2009). Insect OBPs can be divided into five subfamilies: classic, minus-C, plus-C, atypical, and dimer. Classic OBPs possess six conserved cysteines (C_1_–C_6_), paired to form three complete salt bridges (Leal *et al.* 1999). By contrast, minus-C lacks two conserved cysteines (C_2_ and C_5_) (Hekmat-Scafe *et al.* 2002), and plus-C OBPs have two additional conserved cysteines and a proline. Atypical and dimer OBPs are rare; the former possess 12 conserved cysteine residues (Graham and Davies 2002), and the latter have an additional conserved cysteine in an extended C-terminal region, compared with classic OBPs (Xu *et al.* 2003).

In the Lepidoptera, OBPs are divided, according to differences in odorant substrate, into pheromone-binding proteins (PBPs) and general OBPs (GOBPs). Generally, PBPs possess three paralog genes (PBP1, PBP2, and PBP3) and GOBPs contain two (GOBP1 and GOBP2). It was thought that the paralog genes in the PBPs bound pheromone components and those in GOBPs bound general odors (Jacquin-Joly *et al.* 2000; Vogt *et al.* 2015). However, a recent study stated that GOBP2 assists moth larvae to find better food though sex pheromone cues (J. Zhu *et al.* 2016).

In insects, CSPs are more conserved than OBPs (Xu *et al.* 2009). CSPs have two disulfide bonds formed by four conserved cysteines (Angeli *et al.* 1999; Leal *et al.* 1999). The existence of some CSPs in subsets of chemosensory sensilla suggests a potential olfactory function, which has been confirmed by several odor-binding experiments with CSPs (Briand *et al.* 2002; Ozaki *et al.* 2005; González *et al.* 2009; Guo *et al.* 2011). However, CSPs also have nonolfactory functions because of their varied expression patterns, such as being involved in leg regeneration (Kitabayashi *et al.* 1998) and insecticide resistance (G. X. Liu *et al.* 2014). Some OBP and CSP members have been found to be abundant in antennae and enriched in other tissues, such as the female sex PGs (Dani *et al.* 2011; Jacquin-Joly *et al.* 2001). These results strengthen our assertion that OBPs and CSPs do not just play a part in olfaction, but may participate in other crucial physical functions (Sun *et al.* 2012).

The cytochrome P450 monooxygenases (P450s, CYPs) are a large and complex superfamily found across life forms, from prokaryotes to eukaryotes, and are responsible for the oxidative metabolism of many diverse compounds (Nebert and Gonzalez 1987). In insects, the major role of CYPs is to catalyze the synthesis of endogenous, physiologically crucial chemical compounds, such as juvenile hormones, odors, and ecdysteroids. Despite this, most research has focused on their role in the detoxification of pesticides and plant allelochemicals (N. Liu *et al.* 2015). In the fall webworm, *Hyphantria cunea*, one P450 gene (*CYP341B14*) has been characterized and found to be able to epoxidize a specific (Z)-double bond at the ninth position in its type II pheromone precursor: (3Z,6Z,9Z)-3,6,9-henicosatriene (Rong *et al.* 2014).

*Ectropis grisescens* is a major tea pest that has spread to most tea fields in China. It was recently distinguished from its sibling species, *Ectropis obliqua*, by cytochrome oxidase I sequencing (Nan *et al.* 2014; Yu *et al.* 2014). The moth sex pheromone components have been characterized as two type II compounds: Z3, Z6, Z9-18:H and Z3, epo6, Z9-18:H, and they trigger a strong gas chromatography–electroantennogram detection reaction in male moths (Ma *et al.* 2016). Moreover, a 1:4 ratio of the two compounds gives the most attractive effect in tea fields (Ma *et al.* 2016). However, the biosynthesis mechanism for sex pheromone components remains unclear. We aimed to find CYP, OBP, and CSP members potentially involved in sex pheromone biosynthesis and transportation. We sequenced the PG transcriptome of *E. grisescens* and then analyzed the phylogenetic tree and tissue expression patterns of these three gene types.

## Materials and Methods

### Insect samples and tissue collection

The *E. grisescens* colony used in this study was originally collected from the experimental tea plantation in the Tea Research Institute of the Chinese Academy of Agricultural Sciences (Hangzhou, Zhejiang, China). Newly hatched larvae were transferred onto fresh tea shoots in enclosed nylon mesh cages (70 × 70 × 70 cm). They were kept in a climate-controlled room at 25 ± 1° with 70 ± 5% relative humidity under a photoperiod of 14:10 (light:dark). After pupation, male and female pupae were separated based on their body size and morphological characters and kept in darkness until eclosion. After emergence, adult moths were given a 10% honey solution. For transcriptome sequencing, each biological replicate was made up of 40 female PGs from unmated females collected 2–3 d after eclosion. For the quantitative polymerase chain reaction (qPCR), a different sample of 20 female adults was used to collect antennae, proboscises, heads without antennae and proboscises, thoraxes, sex PGs, abdomens without sex PGs, legs, and wings, as well as 20 male adults to collect antennae, proboscises, heads without antennae or proboscises, thoraxes, abdomens, legs, and wings. All tissues were immediately snap-frozen in liquid nitrogen and stored at −80° until extraction. Total RNA was extracted using an SV Total Isolation System (Promega, Madison, WI). The integrity of RNA samples was evaluated by gel electrophoresis and the quantification determined using a NanoDrop 2000 spectrophotometer (NanoDrop, Wilmington, DE).

### cDNA library construction and Illumina sequencing

The cDNA library construction and Illumina sequencing of the samples were performed by Novogene Bioinformatics Technology Co. Ltd., Beijing, China. Poly-adenylated RNA was isolated from 20 µg of the total pooled RNA using oligo (dT) magnetic beads. The mRNA was then fragmented into short pieces in the presence of divalent cations in fragmentation buffer at 94° for 5 min. Using the cleaved fragments as templates, random hexamer primers were used to synthesize first-strand cDNA. Second-strand cDNA was generated using the buffer, dNTPs, RNAse H, and DNA polymerase I. Following end repair and adaptor ligation, short sequences were amplified by PCR and purified with a QIAquick PCR extraction kit (Qiagen, Venlo, The Netherlands), then sequenced on a HiSeq 2000 platform (Illumina, San Diego, CA).

### Assembly and annotation

The PG transcriptomes were assembled *de novo* using the short-read assembly program Trinity (version r20140413p1) based on the paired-end reads. Transcripts larger than 150 bp were compared using BLASTX to existing sequences in the protein databases, including the NCBI NR, NT, KO, Swiss-Prot, PFAM, and KOG. We then used the Blast2GO program for gene ontology (GO) annotation of the transcripts and WEGO software to plot the GO annotation results.

### Analysis of transcript expression in the pheromone glands

Transcript expression abundances were calculated by the FPKM (reads per kilobase per million mapped reads) method, which can eliminate the influence of different transcript lengths and sequencing discrepancies in the calculation of expression abundance. FPKM was calculated using equation (1):

$$FPKM(A)=\frac{C\times 10^{6}}{\frac{N\times L}{10^{3}}}$$ (1)

where FPKM (A) is the expression of transcript *A*; *C* is the number of reads uniquely aligned to transcript *A*; *N* is the total number of fragments uniquely aligned to all transcripts; and *L* is the number of bases in transcript *A*.

### Phylogenetic analysis

To investigate the phylogenetic relationships between the CYPs, OBPs, and CSPs of *E. grisescens* and those of some other insect genes, we compared them using MAFFT with default settings. The phylogenetic tree was constructed using PhyML 3.0 with default settings and 1000 bootstrap replicates.

### Quantitative real-time PCR and data analysis

Quantitative real-time PCR (qRT-PCR) was conducted according to guidelines for the minimum information required for publication of such experiments (Bustin *et al.* 2009). A blank control without template cDNA (replacing cDNA with H_2_O) served as the negative control. Each reaction had three independent biological replicates and was repeated three times (technical replicates). Relative expression levels were calculated using the comparative 2^−△△Cq^ method. Total RNA was isolated using the SV Total Isolation System (Promega, Madison, WI) according to the manufacturer’s instructions, including a step of DNAse treatment to avoid genome contamination. Single-stranded cDNA templates were synthesized using 1 µg of total RNA from 15 moth body and 15 PG samples using a Reverse Transcription System (Promega) following the instructions in the manual. qRT-PCR was performed in a Mastercycler ep realplex (Eppendorf, Hamburg, Germany) with primers designed using Beacon Designer 7.7 and based on the *E. grisescens* nucleotide sequences from the Illumina data. The *E. grisescens GTP-binding protein* and *glyceraldehyde-3-phosphate dehydrogenase* genes were used as references, as they had been identified as having stable expression across tissues in this species. Expression levels of the tested mRNA were determined using GoTaq qPCR Master Mix (Promega) according to the manufacturer’s instructions. The primers are listed in Supplemental Material, File S2.

### Data availability

The datasets of PG transcriptomes used in this study are available in the NCBI SRA database (http://trace.ncbi.nlm.nih.gov/Traces/sra/), under accession numbers SRR5571992; SRR5571993; SRR5571994 and SRR5571995.

## Results

### Overview of the PG transcriptomes

**Figure 1:**
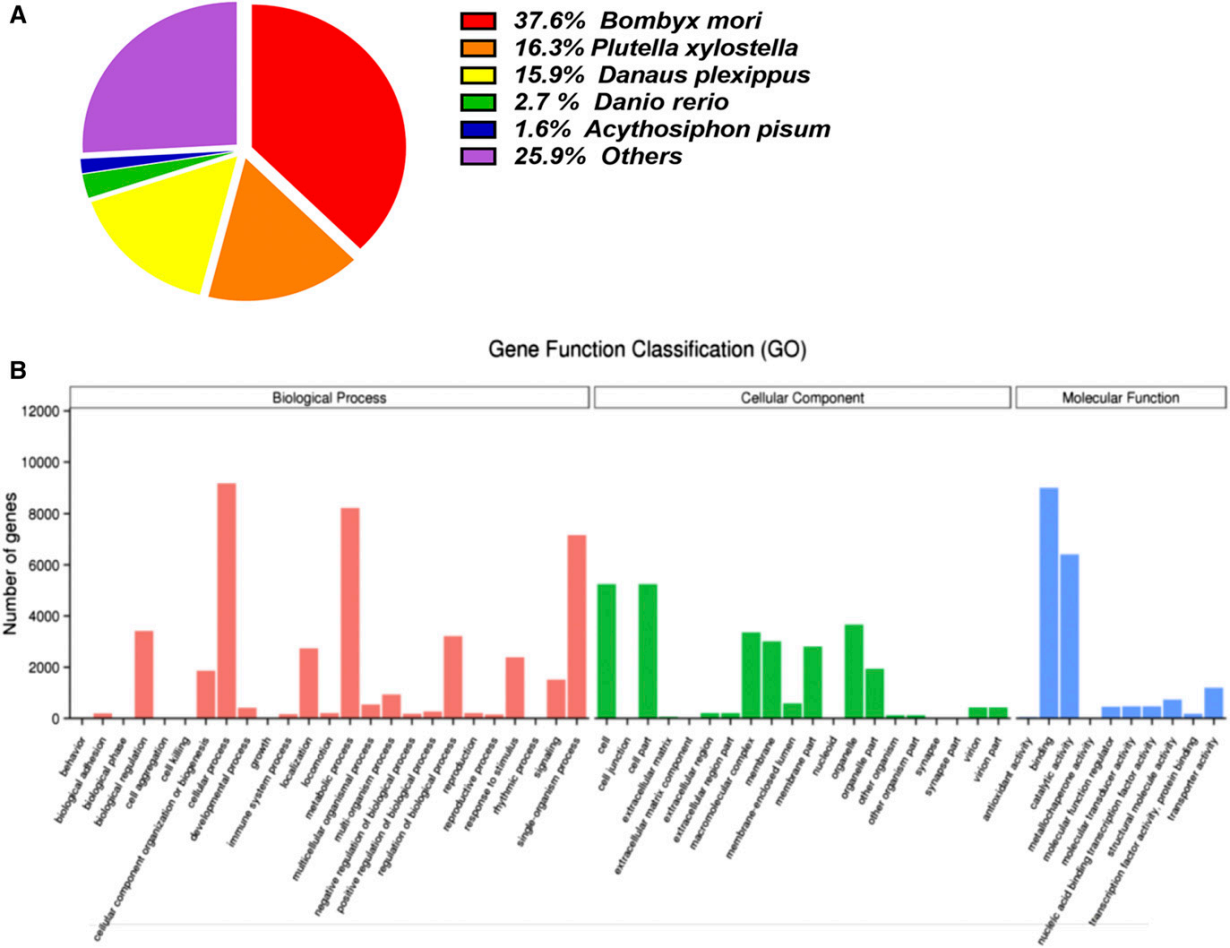
Annotation summaries for *E. grisescens* unigenes. (A) Species distribution of unigenes with the best-hit annotation terms in the nonredundant (NR) database. (B) Gene ontology (GO) classifications of *E. grisescens* unigenes.

The female PG transcriptome was sequenced using the Illumina HiSeq platform and assembled with the program Trinity (version r20140413p1). Two biological replicates were sequenced, yielding 7.31 and 7.41 Gb each. We performed a *de novo* assembly of the PG transcriptomes, yielding 101,632 transcripts with N_50_ lengths of 1491 nt (Figure S1 and Table 1). BLASTx searches of all 76,074 unigenes showed that 28.56% were homologous to proteins in several other insect species. The highest level of sequence identities (37.6%) was with *Bombyx mori* sequences, followed by sequences from *Plutella xylostella* (16.3%), *Danaus plexippus* (15.9%), and *Acythosiphon pisum* (1.6%) (Figure 1A).

**Table 1:** Summary of *Ectropis grisescens de novo* PG transcriptome assembly

	*Ectropis grisescens*	
	PG-1	PG-2
Total Number of Raw Reads	51,184,742	51,899,126
Total Number of Clean Reads	48,724,984	49,372,382
Total Number of Clean Nucleotides (nt)	7.31 Gb	7.41 Gb
Q_20_ Percentage	96.94%	96.83%
GC Percentage	46.64%	46.16%
Total Number of Transcripts	76,074	
N_50_ (nt)	1491	
Percentage of Transcripts Annotated by NCBI NR Database	28.56%	
Percentage of Transcripts Annotated by Swiss-Prot Database	19.05%	
Percentage of Transcripts Annotated by PFAM Database	21.63%	
Percentage of Transcripts Annotated by KOG Database	13.35%	
Percentage of Transcripts Annotated by GO Database	21.88%	

We used Blast2GO to annotate the unigenes into functional groups based on GO. In the molecular function category, the genes expressed were mostly enriched for catalytic activity (*e.g.*, hydrolase and oxidoreductase) and binding (*e.g.*, nucleotide, ion, and odor binding). In the biological process category, cellular and metabolic processes were the most common. In the cellular component category, the terms cell (GO:0005623) and cell part (GO:0044464) were represented the most (Figure 1B).

### Identification of putative OBP, CSP, and CYP genes

To identifiy putative *OBP*, *CSP*, and *CYP* genes, we used a local blast program against the *OBP* and *CSP* genes of *H. cunea* (Zhang *et al.* 2016) and the *CYP* genes of *Operophtera brumata*, since its genome is available and it also belongs to the Geometridae (Derks *et al.* 2015). One hundred and eleven *CYP* transcripts were identified throughout the PG transcriptome, and they showed high levels of identity (from 39.96 to 92.82%) with other insect *CYP* genes (File S1). Helix-C (WxxxR) and helix-K (ExxR), the conservative domains of *CYP* genes, were more conserved than other domains, such as the heme-binding domain (PFxxGxRxCxG/A), the PERF motif (PxxFxPE/DRF), and helix-I (GxE/DTT/S). Additionally, 25 OBPs and 20 CSPs were identified, all of which displayed conservative cysteine domains and high levels of identity with OBPs and CSPs that were previously identified in other insect species: 25–95% for OBPs and 31–90% for CSPs (File S1).

### Phylogenetic tree analysis

**Figure 2:**
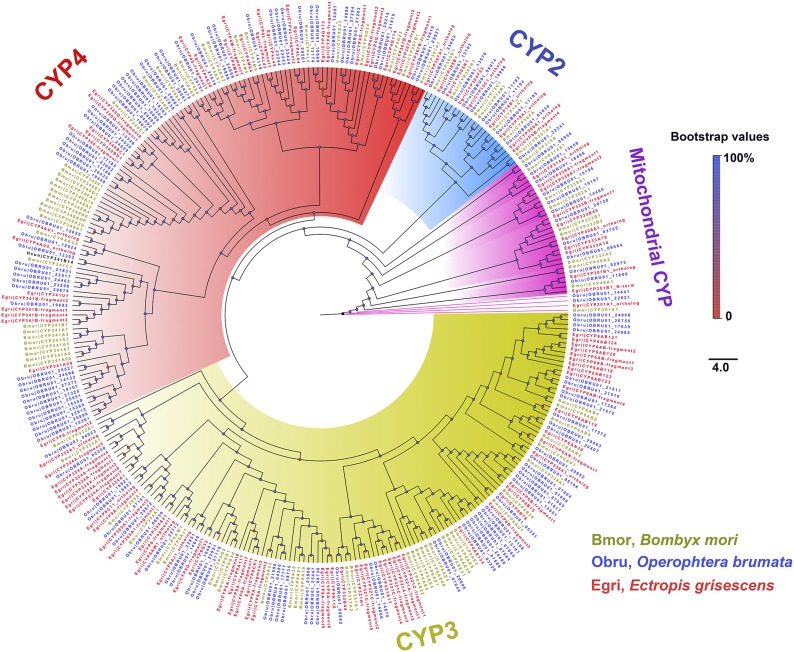
Phylogenetic analysis of CYPs in *E. grisescens*, *O. brumata*, and *B. mori*. The phylogenetic tree was constructed in PhyML3.0 using the maximum likelihood method.

**Figure 3:**
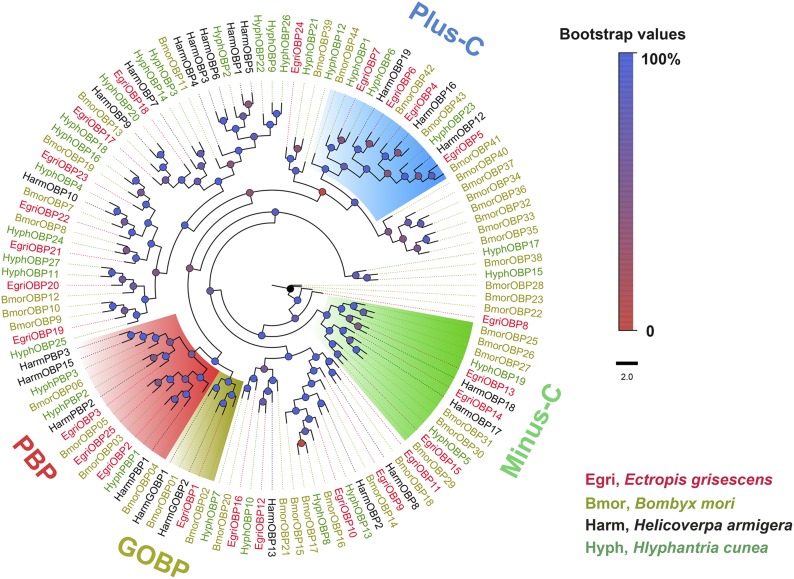
Phylogenetic analysis of EgriOBPs with some other insect OBPs. The phylogenetic tree was constructed in PhyML3.0 using the maximum likelihood method.

**Figure 4:**
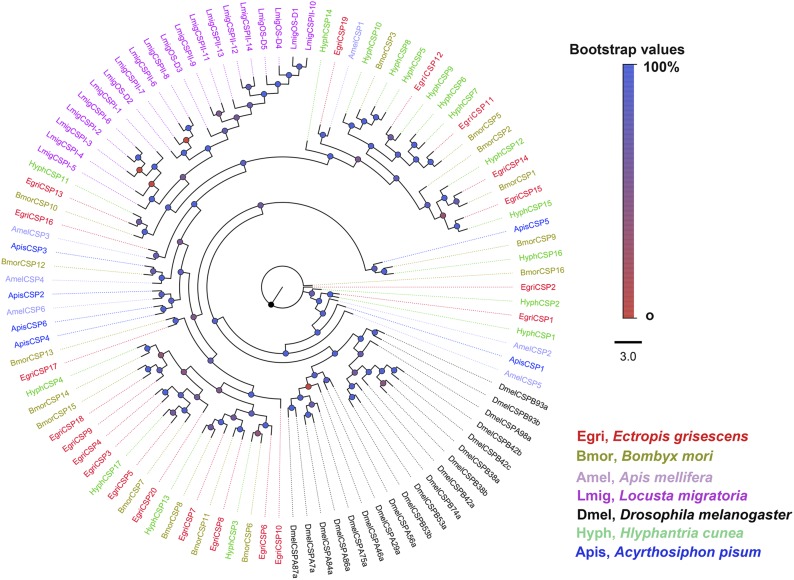
Phylogenetic analysis of EgriCSPs with some other insect CSPs. The phylogenetic tree was constructed in PhyML3.0 using the maximum likelihood method.

*E. grisescens* CYPs were named according to the CYP Gene Family Nomenclature Committee (Dr D. Nelson, University of Tennessee, Memphis, TN). We constructed a phylogenetic tree using *O. brumata* and *B. mori* CYP genes (Figure 2). The 111 CYPs were clearly distributed in all four CYP clans: CYP2, CYP3, CYP4, and mitochondrial CYP. Most of them were distributed in the CYP3 clan (59 genes) and the CYP4 clan (33 genes). The rest were clustered within the CYP2 (7 genes) and mitochondrial CYP (12 genes) clans. For the OBP phylogenetic tree (Figure 3), we used four previously identified groups: plus-C, minus-C, PBP, and GOBP. We found four OBPs in plus-C (EgriOBP4, 5, 6, and 7), three in minus-C (EgriOBP13, 14, and 15), three in PBP (EgriOBP2, 3, and 25), and one in GOBP (EgriOBP1). EgriOBP8 was not clustered in any group, as was the case for several BmorOBPs (22, 23, and 28). For CSPs (Figure 4), one group included the most (10) EgriCSPs (EgriCSP3, 4, 5, 6, 7, 8, 9, 10, 17, and 18), as well as CSPs from the two other lepidopteran species: seven from *B. mori* and four from *H. cunea*. Another group contained only lepidopteran CSPs and one *Apis mellifera* CSP: AmelCSP1. In this group, there were five EgriCSPs: 11, 12, 14, 15, and 19. EgriCSP13 and its orthologous genes, BmorCSP10 and HyphCSP11, clustered within a clade of *Locusta migratoria* CSPs. EgriCSP2 did not cluster in any group, and another 19 CSPs were distributed in four other groups.

### Tissue expression profile and mRNA abundance of the CYP, OBP, and CSP genes

**Figure 5:**
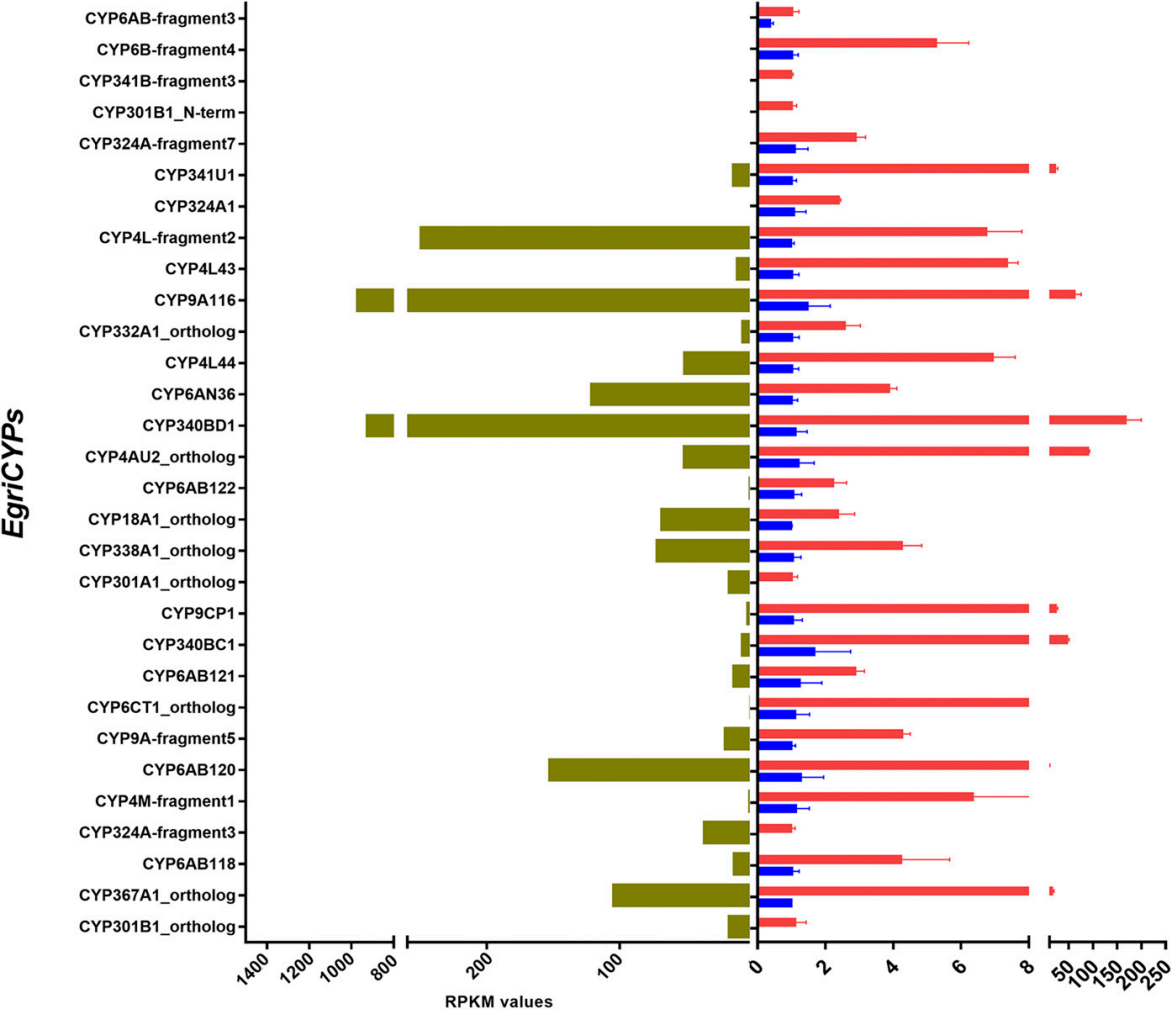
Tissue expression profile of selected *EgriCYP* genes based on relative mRNA quantity and the FPKM values of EgriCYPs with PG-enriched expression. The level of *EgriCYPs* expression in the abdomen was set at 1. B, abdomen; Pg, female pheromone glands.

We further characterized the expression levels and tissue expression patterns of the transcripts of the putative *CYP*, *OBP*, and *CSP* genes by qPCR. The aim was to find the genes with expression predominantly in the PG, which may be involved in pheromone biosynthesis or transportation. Transcript abundance in the PG was also calculated as FPKM (File S1). One hundred of the 111 CYPs were successfully amplified by qPCR (Figure 5). Of them, 30 CYPs presented a PG-enriched expression pattern. The mRNA abundance showed that seven *EgriCYPs* had expression levels >10 times higher in the PG than in the body: *EgriCYP340BD1* (149.05-fold), *EgriCYP367A1_Ortholog* (74.16-fold), *EgriCYP4AU2* (42.53-fold), *EgriCYP9A116* (28.16-fold), *EgriCYP340BC1* (22.58-fold), *EgriCYP9CP1* (22.54-fold), and *EgriCYP341U1* (16.12-fold). Five of the 100 CYPs were clearly expressed at higher levels than the rest: *EgriCYP9A116* (FPKM = 940.07 ± 98.71), *EgriCYP340BD1* (929.68 ± 0.71), *EgriCYP4L-fragment2* (250.21 ± 9.52), *EgriCYP4G174* (202.27 ± 33.27), and *EgriCYP6AB120* (153.37 ± 10.59) (Figure 5).

**Figure 6:**
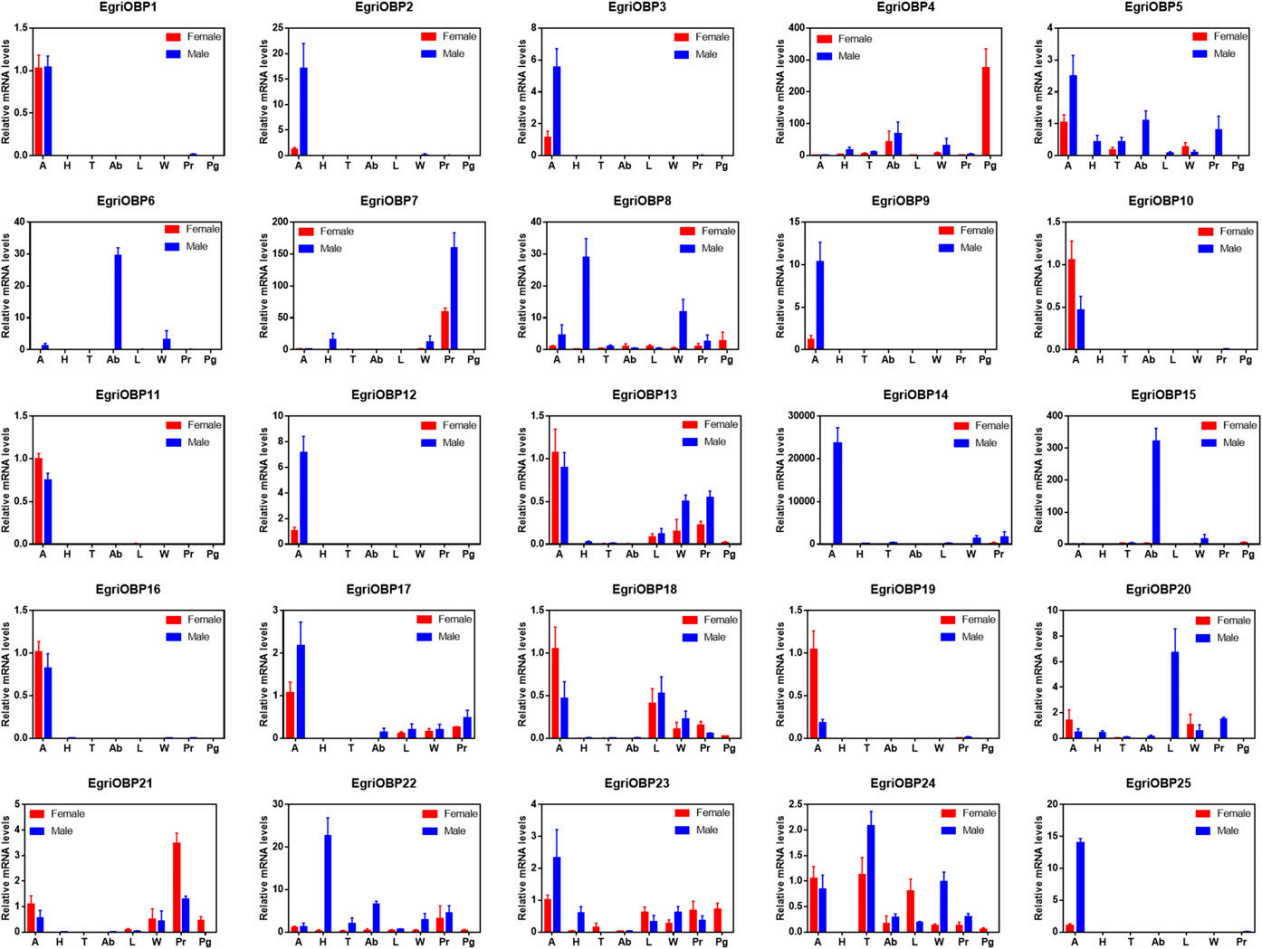
Tissue expression profile of selected *EgriOBP* genes based on relative mRNA quantity. The level of *EgriOBPs* expression in the female antennae was set at 1. A, antennae; Ab, abdomen; H, heads; L, legs; Pg, female pheromone glands; Pr, proboscises; T, thoraxes; W, wings.

For the *OBPs* (Figure 6), 25 *EgriOBPs* presented distinct expression patterns. The *OBPs* with greater levels of expression in male antennae (*EgriOBP2*, *3*, *5*, *9*, *12*, *14*, and *25*) had ratios of male:female antennae expression of 14.42, 4.79, 2.37, 8.51, 6.73, 4184.72, and 13.28, respectively. Five *OBPs* were expressed equally in male and female antennae (*EgriOBP1*, *11*, *13*, *16*, and *17*). Only one *OBP* (*EgriOBP4*) presented a female PG-enriched expression pattern, with its expression level in the PG being at least four times higher than that in other tissues. Additionally, three *OBPs* (*EgriOBP10*, *18*, and *19*) showed a pattern of abundant expression in female antennae, with the ratios of female:male antennae being 2.25, 2.26, and 5.64, respectively. Of the other OBPs, *EgriOBP8* and *22* were highly expressed in male heads, *EgriOBP6* and *15* had enriched expression in the male abdomen, *EgriOBP20* presented predominant expression in the male leg, and *EgriOBP7* and *21* were highly expressed in proboscises. Two *OBPs* (*EgriOBP23* and *24*) had ubiquitous expression patterns, with no significant differences between the tissues tested.

**Figure 7:**
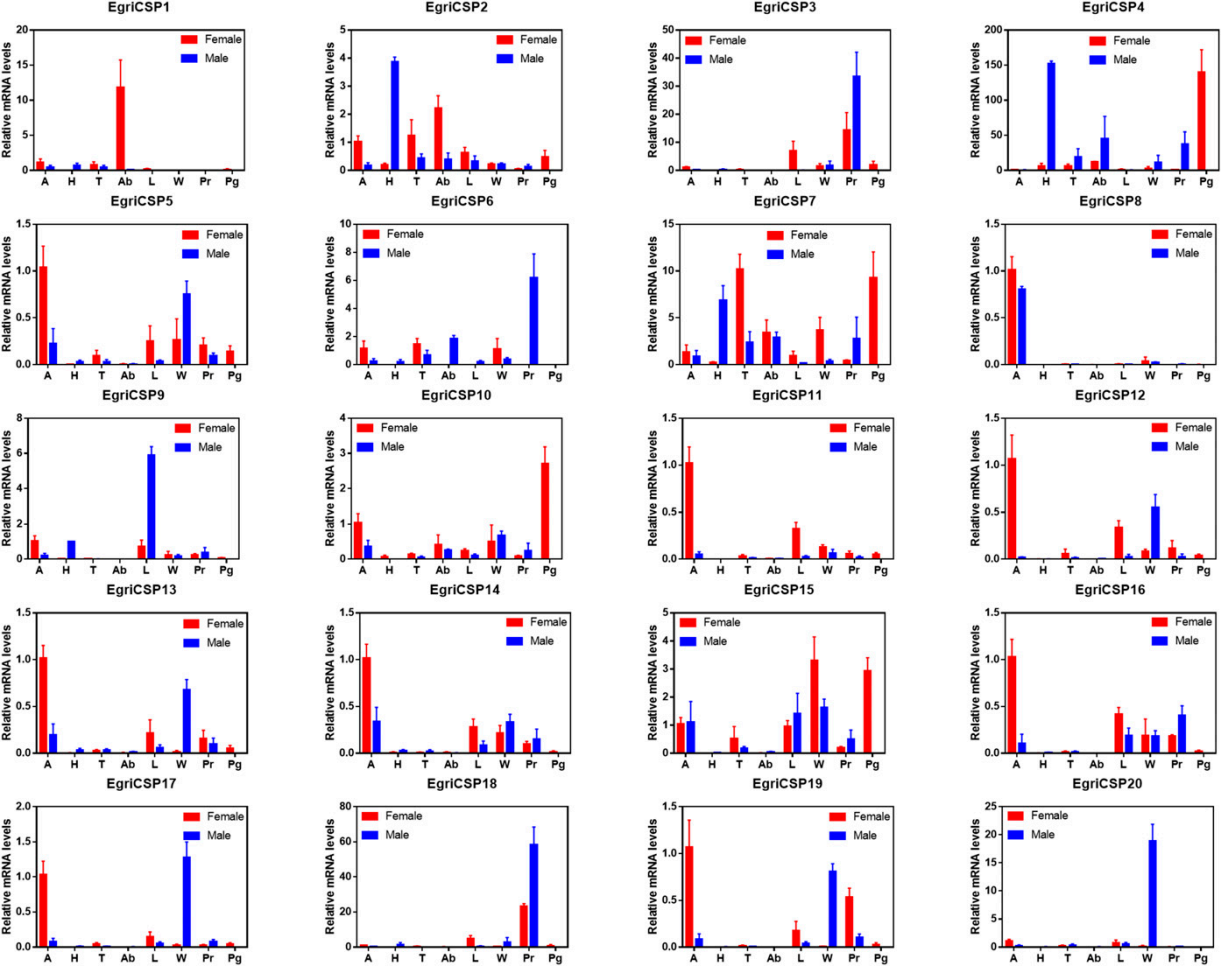
Tissue expression profile of selected *EgriCSP* genes based on relative mRNA quantity. The level of *EgriCSPs* expression in the female antennae was set at 1. A, antennae; Ab, abdomen; H, heads; L, legs; Pg, female pheromone glands; Pr, proboscises; T, thoraxes; W, wings.

As for *CSPs*, seven *EgriCSPs* (*5*, *8*, *11*, *12*, *13*, *14*, and *16*) were found to be expressed at high levels in the antennae. *EgriCSP5*, *11*, *12*, *13*, *14*, and *16* showed dominant expression in the female antennae, which was above their expression levels in other tissues that were tested, whereas *EgriCSP8* showed equal levels in both sexes (Figure 7). Two other *EgriCSPs*, *EgriCSP3* and *18*, showed a proboscis expression pattern indicating their gustatory function. Furthermore, *EgriCSP9*, *10*, and *20* were expressed at obviously raised levels in legs, PGs, and wings, respectively. No other *EgriCSPs* had significantly raised levels of expression in any of our tissues tested. Among the 20 *EgriCSPs*, *EgriCSP5*, *13*, *15*, *17*, and *19* ranked as having the top five expression levels (Figure S2).

## Discussion

The sex pheromone components of *E. grisescens*, Z3, Z6, Z9-18:H and Z3, epo6, Z9-18:H (Ma *et al.* 2016), belong to the type II group. It is expected that triene, a type II pheromone component, is synthesized in epidermal oenocytes before being transported to the PG, where it is oxidized to epoxydiene and then emitted into the atmosphere. We were unable to dissect and extract RNA from oenocytes; instead, we selected PG, since it is an important tissue responsible for the synthesis and release of type I sex pheromones. CYP might be involved in this oxidation process (Rong *et al.* 2014). OBP and CSP are thought to have a transportation function for sex pheromones in the type I group (Liu *et al.* 2013; Zhang *et al.* 2014; Chang *et al.* 2015; Z.-Q. Li *et al.* 2015; N. Y. Liu *et al.* 2015; G.-H.Zhu *et al.* 2016) and may, therefore, also participate in the transportation of type II sex pheromone components.

Our study aimed to identify the genes that are potentially involved in the biosynthesis (CYPs) and transportation (OBPs and CSPs) of type II sex pheromone components. We constructed a massive PG transcriptome database, yielding 14.72 Gb from two biological replicates of *E. grisescens*. The result was 111 CYPs in total. Twenty-five of the OBPs and 20 of the CSPs were first identified in this species. We further analyzed the phylogenetic and tissue expression patterns and found that 30 *EgriCYPs*, *EgriOBP4*, and *EgriCSP10* had enriched expression patterns in the PG, and that EgriOBP2, 3, and 25 clustered in the moth PBP clade. We therefore considered these our candidate genes.

CYP is involved in the epoxidation of triene to epoxydiene in the PGs. It follows that CYPs that are distinctly expressed at higher levels in PGs than in adult somatic cells, and that are more abundant than the other CYPs in the PGs, may be involved in sex pheromone biosynthesis. Five *CYPs* displayed PG-predominant expression, had far higher abundance than other *CYPs* in the PGs: *EgriCYP9A116*, *EgriCYP340BD1*, *EgriCYP4L-fragment2*, *EgriCYP4G174*, and *EgriCYP6AB120*. Therefore, these five *CYP* genes might function in sex pheromone biosynthesis. However, previous research stated that in the fall webworm *H. cunea*, one CYP member (CYP341B14) was able to epoxidize a specific (Z)-double bond in a pheromone precursor, (3Z,6Z,9Z)-3,6,9-henicosatriene (Rong *et al.* 2014). One of the type II sex pheromone components in *E. grisescens* is Z3, epo6, Z9-18:H, which contains an epoxide group at the sixth position. We found that its orthologous gene is also a CYP341 member (*EgriCYP341B-fragment3*) with a dominant expression pattern in the PG. It showed high levels of identity (77%) with CYP341B14 of *H. cunea*. Further studies will focus on full-length cloning and functional characterization of these six *CYP* genes.

The phylogenetic tree analysis of *EgriOBPs* showed that EgriOBP2, 3, and 25 cluster in a moth PBP clade. Tissue expression pattern results showed that the three *OBPs* highly expressed in male antennae correspond to expression patterns in other moth PBPs (Liu *et al.* 2013; Sun *et al.* 2013; Jin *et al.* 2014; N. Y. Liu *et al.* 2015). Notably, we found two PBP1 analogs (*EgriOBP2* and *25*), and one PBP2 analog (*EgriOBP3*), but no PBP3 analog. Another moth, *H. cunea*, has the miscellaneous pheromone type and full PBP1, 2, and 3 genes (Zhang *et al.* 2016). It may be that the moths with type II pheromones and PBP3 genes have not separated from PBP1. Only the female PG transcriptome database has been built, so the antennae transcriptome and genome of this species is needed to further confirm our results and conclusions. Similarly, in the GOBP clade, GOBP1 could not be found in our database; only the GOBP2 analog was identified. However, among 25 *EgriOBPs*, the mRNA expression of four genes (*EgriOBP1*, *2*, *3*, and *25*) did not rank highly among all *EgriOBPs*. There are some olfactory sensilla distributed on the ovipositor (Faucheux 1988). *EgriOBP4* is the only OBP gene to display a PG-enriched expression pattern, which ranked it sixth in expression level of the OBPs in the PG. We propose that, in *E. grisescens*, *EgriOBP2*, *3*, *4*, and *25* are type II sex pheromone transporters and that further functional characterization is needed.

Unlike the *EgriOBPs*, the expression patterns of *EgriCSPs* are similar to those of other insect CSPs (Z.-Q. Li *et al.* 2015; X.-M. Li *et al.* 2015; Zhang *et al.* 2016). Interestingly, we did not find any male antennae with enriched CSP expression levels, but found three female antennae enriched with *EgriCSPs*: *5*, *11*, *12*, *14*, and *16*. These may participate in female-specific physiological behaviors in moths, such as locating ovipositor sites. We found high levels of *EgriCSP3* and *18* expression in proboscises, suggesting that they may have a role in moth tasting, as was found in previous functional characterizations of CSPs from *Helicoverpa armigera* and *Helicoverpa assulta* (Y. L. Liu *et al.* 2014). Three BmorCSPs (6, 11, and 15) of *B. mori* were detected by protein sequencing in PGs but not in antennae (Dani *et al.* 2011). Their analogs are *EgriCSP6*, *8*, and *18*, respectively, but these are not expressed at high levels in PGs. In this study, *EgriCSP10* was the only *EgriCSP* expressed at high levels in PGs, and we thus propose that they are involved in sex pheromone transportation in this moth.

## Supplementary Material

Supplemental material is available online at www.g3journal.org/lookup/suppl/doi:10.1534/g3.117.300543/-/DC1.
