# Supplementary figures and images for: Gene Identification of Pheromone Gland Genes Involved in Type II Sex Pheromone Biosynthesis and Transportation in Female Tea Pest Ectropis grisescens
Source: G3 (Bethesda). 2018 Jan 9;8(3):899–908. doi: 10.1534/g3.117.300543 (PMC5844310; doi:10.1534/g3.117.300543)

**Figure S1**

**
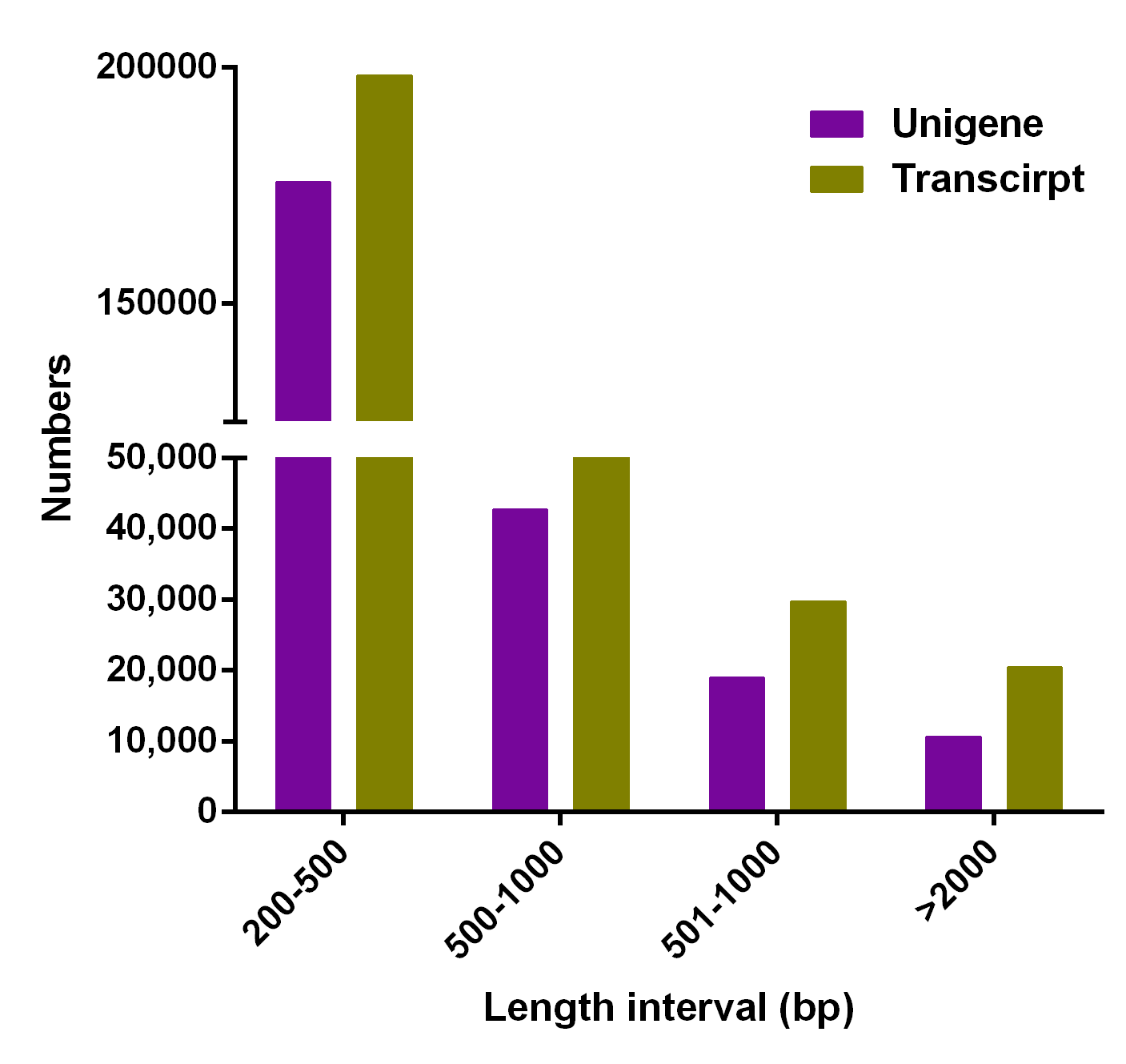
**

Supplement: Supplementary file 1 [file 899FigureS1.doc]

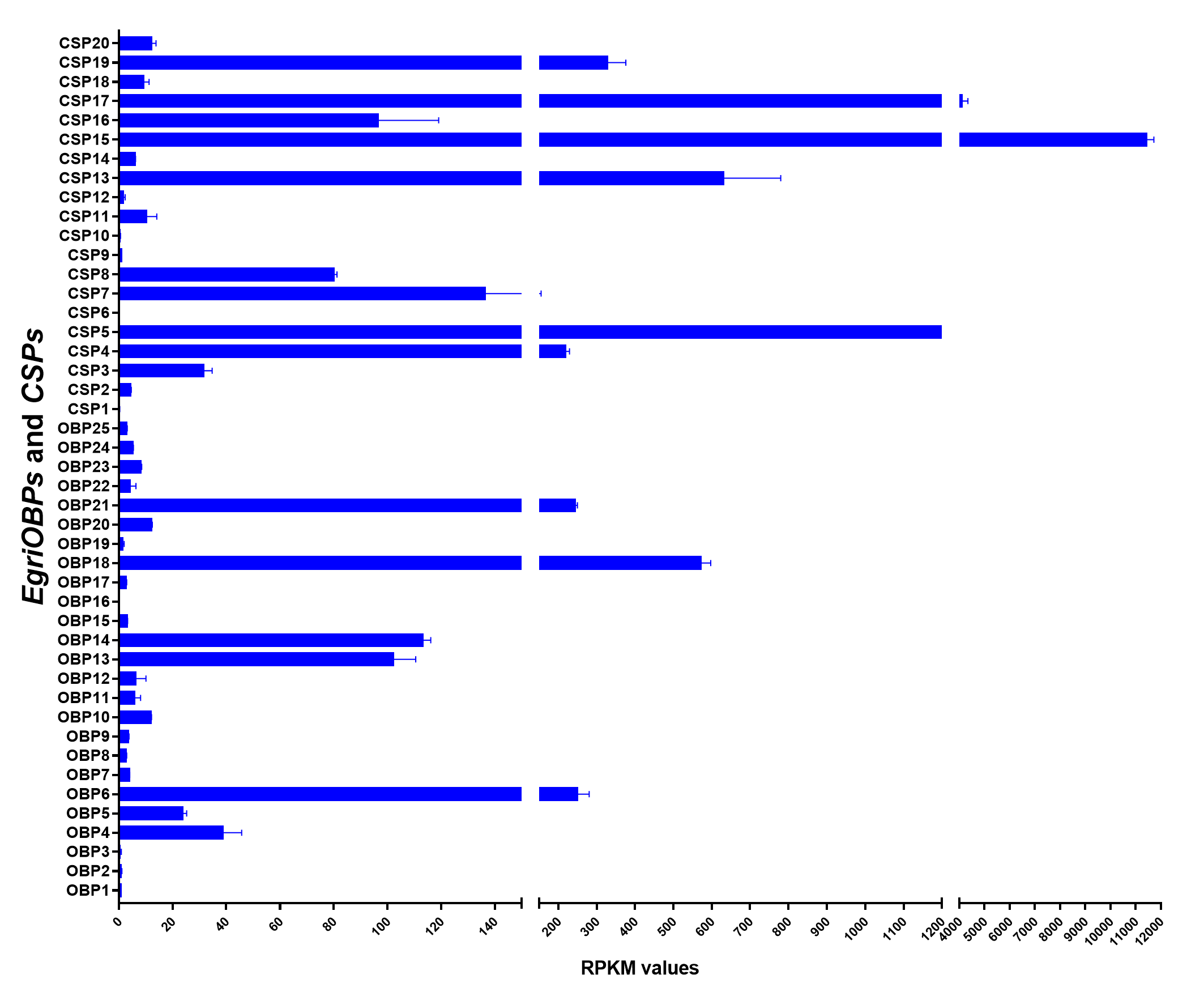

Supplement: Supplementary file 2 [file 899FigureS2.tif]
